# Next-Generation HER2-Targeted Antibody–Drug Conjugates in Breast Cancer

**DOI:** 10.3390/cancers16040800

**Published:** 2024-02-16

**Authors:** Brittney S. Zimmerman, Francisco J. Esteva

**Affiliations:** 1 Northwell, New Hyde Park, NY 11042, USA; bzimmerman2@northwell.edu; 2 Northwell Health Cancer Institute, Lake Success, NY 11042, USA

**Keywords:** HER2, antibody–drug conjugates, breast neoplasms, targeted therapy, trastuzumab deruxtecan, trastuzumab-DM1, drug resistance

## Abstract

**Simple Summary:**

Human epidermal growth factor receptor 2 (HER2) is amplified or overexpressed in approximately 20% of breast cancer cases. This overexpression is correlated with a more aggressive form of the disease and predicts a less favorable prognosis. Historically, the standard treatment for patients with HER2-positive breast cancer has involved chemotherapy in combination with monoclonal antibodies that target the HER2 receptor, notably trastuzumab and pertuzumab. However, resistance to these drugs has been ubiquitous, presenting challenges in the management of the disease. Antibody–drug conjugates (ADCs) represent a promising therapeutic category for the treatment of breast cancer. Trastuzumab emtansine (T-DM1) and trastuzumab deruxtecan (T-DXd) are currently approved ADCs. These molecules have been pivotal, demonstrating superior clinical outcomes over previous conventional treatments in HER2-positive breast cancer (T-DM1, T-DXd) and HER2-Low (T-DXd). However, drug resistance remains an unresolved problem, and there is interest in developing better-tolerated and more effective therapeutics for breast cancer. This review focuses on the emergence of innovative HER2-targeted ADCs, including those currently undergoing investigation in clinical trials.

**Abstract:**

Human epidermal growth factor receptor 2 (HER2) tyrosine kinase is overexpressed in 20% of breast cancers and associated with a less favorable prognosis compared to HER2-negative disease. Patients have traditionally been treated with a combination of chemotherapy and HER2-targeted monoclonal antibodies such as trastuzumab and pertuzumab. The HER2-targeted antibody–drug conjugates (ADCs) trastuzumab emtansine (T-DM1) and trastuzumab deruxtecan (T-DXd) represent a novel class of therapeutics in breast cancer. These drugs augment monoclonal antibodies with a cytotoxic payload, which is attached by a linker, forming the basic structure of an ADC. Novel combinations and sequential approaches are under investigation to overcome resistance to T-DM1 and T-DXd. Furthermore, the landscape of HER2-targeted therapy is rapidly advancing with the development of ADCs designed to attack cancer cells with greater precision and reduced toxicity. This review provides an updated summary of the current state of HER2-targeted ADCs as well as a detailed review of investigational agents on the horizon. Clinical trials are crucial in determining the optimal dosing regimens, understanding resistance mechanisms, and identifying patient populations that would derive the most benefit from these treatments. These novel ADCs are at the forefront of a new era in targeted cancer therapy, holding the potential to improve outcomes for patients with HER2-positive and HER2-Low breast cancer.

## 1. Introduction

Breast cancer is the most common malignancy among women and a leading cause of cancer-related deaths worldwide [1]. There are three main molecular subtypes of breast cancer, which differ significantly in natural history, prognosis, and treatment options. These are commonly known as hormone receptor-positive/HER2-negative (luminal A, luminal B), human epidermal growth factor receptor 2 (HER2)-positive, and triple-negative [2]. The HER2 protein is overexpressed in 20–30% of breast cancers and is historically associated with higher cancer recurrence rates and shorter disease-free and overall survival when compared with HER2-negative breast cancers [3]. HER2 is a member of the epidermal growth factor receptor (EGFR) family of receptor tyrosine kinases, which includes EGFR/HER1, HER2, HER3, and HER4. These receptors play a significant role in cell growth and differentiation. Overexpression of HER2 is linked to upregulated cell proliferation and the development of several cancers, including breast, gastric, and ovarian cancer [3,4]. Identification of the HER2 status is achieved by two methods: immunohistochemistry (IHC) and fluorescence in situ hybridization (FISH). All patients with invasive breast cancer have the tumor evaluated first by IHC (graded from 0 to 3+), followed by validation with FISH for borderline (IHC 2+) results. Patients with tumors that are IHC 3+- or FISH-positive tend to respond to anti-HER2 therapies. HER2 testing methodologies have evolved over time, including different ways of assessing HER2 protein expression using immunohistochemistry with novel scoring systems (e.g., H-score). HER2-Low breast cancer represents a new subset of breast cancers that may respond to HER2 ADC therapy. The development and validation of quantitative assays to determine HER2 protein levels in breast cancer tissue samples is an area of active investigation [5].

The approval of trastuzumab, a monoclonal antibody targeting HER2, in 1998 marked a significant advancement in the therapeutic landscape for HER2-positive breast cancer [6]. Subsequently, there has been a substantial increase in the clinical development of innovative therapies aimed at this specific breast cancer subtype [7]. The strategic incorporation of monoclonal antibodies and tyrosine kinase inhibitors, along with chemotherapy, endocrine therapy, and immunotherapy, has profoundly transformed the clinical outcomes for individuals diagnosed with HER2-positive breast cancer [8]. This paradigm shift has occurred at both the early and advanced stages of the disease [9].

While monoclonal antibodies and tyrosine kinase inhibitors represent major advances in the treatment of HER2-positive breast cancer, a significant number of patients with early breast cancer develop metastatic disease despite adjuvant systemic therapy. Furthermore, the development of progressive disease in the metastatic setting and the lack of curative therapies remain unmet needs. Understanding molecular mechanisms of resistance in HER2-positive breast cancer cells, together with improvements in technology, led to the development of antibody–drug conjugates (ADCs). These drugs represent an advanced modality in oncological treatment, integrating the specificity of monoclonal antibodies with the cytotoxic power of potent drugs, also known as “payloads”, using specific linkers [10]. As targeted therapeutics, ADCs are designed to selectively hone in on and neutralize cancer cells expressing antigens, specifically the HER2 antigen (for purposes of this review). This targeted approach amplifies treatment efficacy while minimizing the widespread adverse effects typically associated with conventional chemotherapy.

**Figure 1:**
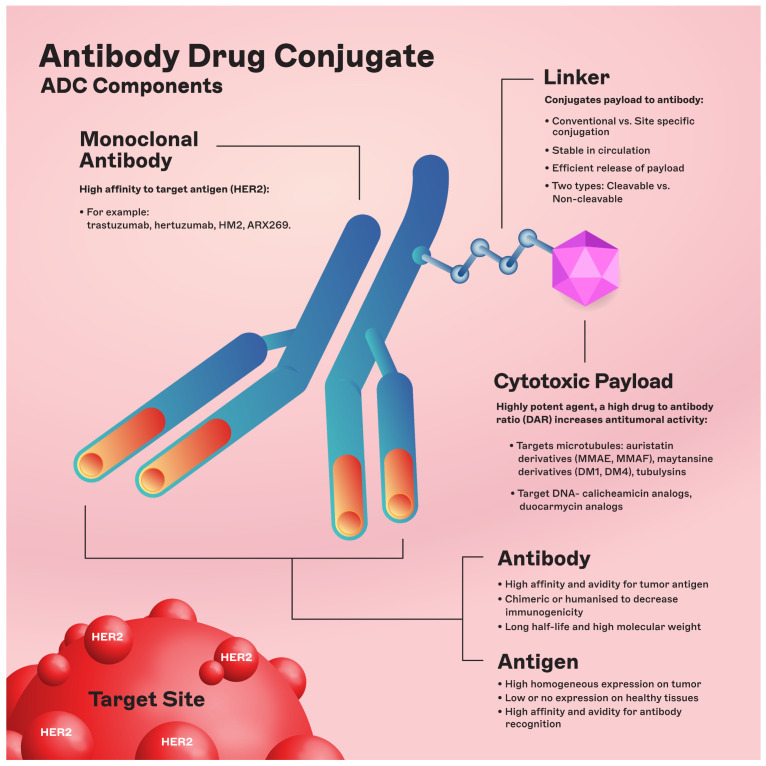
ADC components. Abbreviations: DAR: drug-to-antibody ratio; DM1: derivative of maytansine 1; DM4: derivative of maytansine 4; MMAF: monomethyl auristatin F; MMAE: monomethyl auristatin E.

The chemical structure of ADCs consists of three integral elements: a monoclonal antibody, a chemical linker with stability or cleavability properties, and a cytotoxic payload/agent (Figure 1).

**Figure 2:**
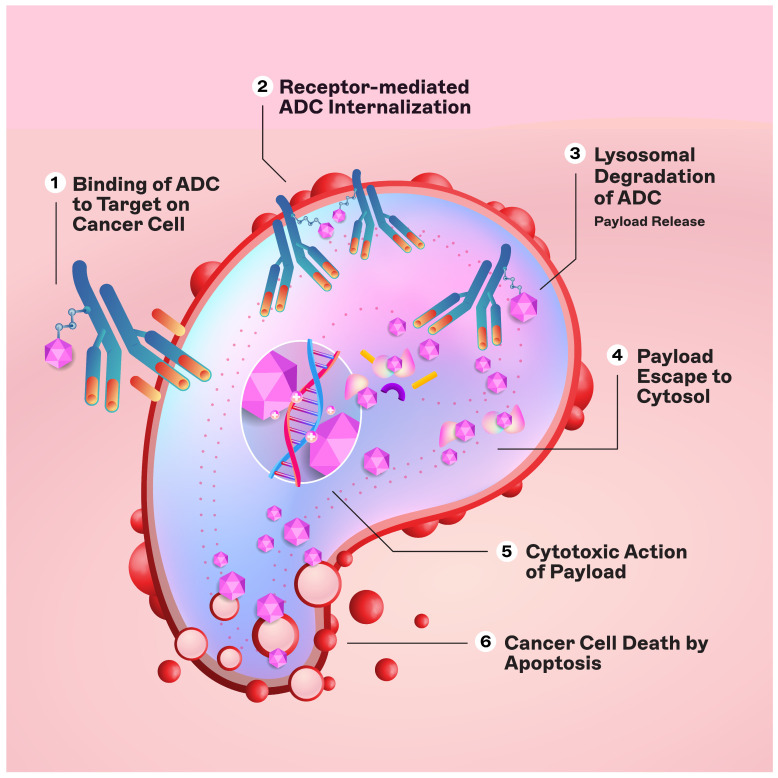
Mechanism of action of ADCs.

The engineered monoclonal antibody (e.g., trastuzumab) is designed to detect and bind to an antigen expressed on the surface of cancer cells (i.e., HER2) [11]. Following attachment, the antibody–drug complex is internalized via receptor-mediated endocytosis [12]. While pinocytosis may also facilitate ADC uptake in the absence of the target antigen, the conjugated antibody’s considerable size and hydrophilic character significantly mitigate nonspecific absorption, thereby augmenting the specificity and safety of ADCs [13]. Upon cellular entry, the ADC is trafficked to endosomes and lysosomes, where enzymatic cleavage of the linker ensues, resulting in the release of the cytotoxic payload. This release enables the drug to unleash its cell-killing potential (Figure 2). The therapeutic agents employed in ADCs are diverse, ranging from microtubule disruptors to topoisomerase inhibitors. A pivotal feature of ADCs is the “bystander effect,” wherein the liberated toxins can permeate and exterminate neighboring tumor cells that may not express the target antigen, thereby increasing the antitumor effect [14].

## 2. Current Landscape of HER2-Targeted ADCs

To date, two ADCs directed against HER2—trastuzumab emtansine (T-DM1, Kadcyla) [15] and trastuzumab deruxtecan (T-DXd, DS-8201a, Enhertu) [16]—have been approved by the Food and Drug Administration (FDA) and other regulatory agencies throughout the world for the management of HER2-positive breast cancer. In addition, T-DXd was approved for patients with HER2-Low metastatic breast cancer (IHC score 1 or IHC 2 with negative FISH) (Table 1) [17]. The DESTINY-Breast06 trial is testing the efficacy of T-DXd in patients with HER2-Ultra-Low breast cancer (IHC score 0, with 1–10% cells staining weakly).

### 2.1. Trastuzumab Emtansine (T-DM1)

Trastuzumab emtansine (T-DM1) represents a seminal advancement in the treatment of HER2-positive breast cancer. T-DM1 (Kadcyla) was the first ADC to receive FDA approval in the U.S. in 2013 for single-agent treatment for advanced HER2-positive breast cancer (following treatment with trastuzumab and a taxane). Its approval was expanded in 2019 for the use of T-DM1 for patients with early-stage high-risk HER2-positive breast cancer with residual disease after neoadjuvant therapy with trastuzumab and taxane-based treatment [19].

The T-DM1 molecule consists of a cytotoxic component (Emtansine) attached to the antibody trastuzumab through a stable linker. Maytansine is a highly potent cytotoxic agent derived from the Ethiopian plant Maytenus serrata. Due to its high toxicity, it is not used directly as a cancer treatment but rather as a part of a targeted therapy. Emtansine (also known as DM1) is a derivative of maytansine that has been chemically modified to be less toxic and more stable in the bloodstream. T-DM1 retains trastuzumab’s inhibitory functions—especially the blockade of the PI3K/AKT pathway. In addition, trastuzumab facilitates T-DM1′s internalization and subsequent disintegration to unleash the potent microtubule-inhibitory action of the MCC-DM1 complex [27].

The EMILIA trial showcased the superiority of T-DM1, demonstrating its ability to improve progression-free survival (PFS) rates compared to a regimen that combined lapatinib and capecitabine in patients with HER2-positive metastatic breast cancer who had received a prior taxane [15]. In this trial, median progression-free survival was improved from 6.4 months with lapatinib–capecitabine to 9.6 months with T-DM1. Overall survival and objective response rates were also improved with T-DM1. The KATHERINE trial showed improvement in disease-free survival (DFS) rates in patients with early-stage HER2-positive breast cancer with residual disease following neoadjuvant trastuzumab-based treatment [19]. At a follow-up of 3 years, the percentage of patients who were free of invasive disease was 88.3% in the T-DM1 group compared with 77.0% in the trastuzumab group. This randomized trial validated the role of T-DM1 for patients with residual disease after neoadjuvant therapy when compared to continued trastuzumab therapy.

Furthermore, in the KRISTINE trial, T-DM1 therapy was compared to sequential anthracycline-based chemotherapy followed by taxane in combination with trastuzumab and pertuzumab, or the TCHP (docetaxel, carboplatin, trastuzumab, pertuzumab) regimen in the neoadjuvant setting. In this study, T-DM1 demonstrated a reduced pathologic complete response rate compared to the other regimens [28]. Nevertheless, the KRISTINE trial linked it to a more favorable safety profile, achieving pathologic complete responses in 44% of patients without conventional chemotherapy.

#### 2.1.1. Mechanisms of Resistance to T-DM1

The mechanisms of resistance to T-DM1 in breast cancer are multifactorial and occur via various different pathways.

1. Antigen-Related Resistance Mechanisms. T-DM1 resistance can develop via various pathways, particularly in JIMT1 cells, which are inherently resistant to first-line trastuzumab [29]. These cells, upon TM-ADC treatment, showed resistance while remaining sensitive to other chemotherapeutics. It suggests that prolonged exposure to HER2-targeted therapy could decrease HER2 levels, leading to treatment-resistant cells [30]. Additionally, heterogeneity in HER2 expression, as observed in the KRISTINE and ZEPHIR trials, correlated with lower efficacy of T-DM1, marked by no pathologic complete responses and shorter progression-free survival (PFS) and overall survival (OS) [31,32]. Truncated forms of the antigen ectodomain, like P95HER2, and antigen masking by molecules such as MUC4 have also been implicated in resistance [33]. Furthermore, ligand-induced heterodimerization of HER2 with other receptors can impair T-DM1’s effectiveness [29].
2. Payload-Related Resistance. Tumor cells may develop resistance to the cytotoxic drug (DM1). T-DM1-resistant cells with upregulated ABC transporter expressions (ABCC2, ABCG2) exhibited reduced sensitivity, which could be countered by inhibiting these transporters [34]. The diversity in payloads, conjugation sites, and drug-to-antibody ratios (DAR) also significantly impacts ADC efficacy, suggesting that ADC resistance can be payload specific [35,36].
3. Internalization and Trafficking Pathways. T-DM1 is internalized into cancer cells via endocytosis. Variations in endocytic routes, regulated by specific proteins, can affect ADC delivery and processing. For instance, some T-DM1-resistant cells have been shown to internalize ADCs into caveolin-1-coated vesicles, indicating an alternative trafficking pathway. Proteins like Endophilin A2 also play a role in HER2 internalization, affecting T-DM1 sensitivity [37].
4. Lysosomal Dysfunction. After T-DM1 internalization, lysosomal cleavage releases the cytotoxic drug. Any disruption in lysosomal function, such as altered pH or proteolytic activity, can hinder this process. Resistant clones with higher lysosomal pH and accumulated T-DM1 have been documented, indicating impaired ADC processing [38]. The transport of cytotoxic drugs from lysosomes to the cytoplasm, especially relevant for non-cleavable linkers, is another potential resistance mechanism [39]. In the DAISY trial, a phase II multicenter, open-label study, researchers investigated resistance to T-DXd in three distinct patient groups: HER2-positive, HER2-zero, and low-HER2. Participants received T-DXd at 5.4 mg/kg triweekly, aiming for the best objective response rate as the primary measure of success. When the cancer progressed, whole-genome sequencing was employed to uncover potential resistance mechanisms. The findings indicated that apart from reduced HER2 expression, alterations in the SLX4 gene could contribute to resistance. SLX4 is integral to DNA damage repair, overseeing three endonucleases. The evidence showed that a deficit in SLX4 correlated with resistance to T-DXd, suggesting that loss-of-function mutations in SLX4 could be implicated in the development of resistance to the drug [40].
5. Drug-Efflux Mechanisms. Overexpression of ABC transporters, which increase drug efflux from cells, can contribute to resistance. For example, maytansinoids, T-DM1’s payload, are known substrates of ABC transporters like MDR1, linking resistance to increased expression of these transporters [41].
6. Cell Cycle Dependencies. The cell cycle status affects T-DM1 effectiveness. Resistance to T-DM1 has been linked to variations in cyclin B levels, a cell cycle regulator. Accumulation of cyclin B1 in sensitive cells, as opposed to resistant ones, suggests that cell cycle dysregulation can modulate T-DM1 efficacy [42].
7. Activation of Survival Signaling Pathways. Activation of pathways like PI3K/AKT/mTOR, which are involved in cell survival, can decrease sensitivity to trastuzumab-based therapy. PTEN loss or PIK3CA hyperactivation can lead to reduced trastuzumab sensitivity [43]. However, T-DM1 may remain effective even with these mutations, as indicated by the EMILIA trial results [44]. Therefore, this is an area of ongoing investigation.
8. Apoptosis Dysregulation. Finally, changes in apoptosis regulation, such as the overexpression of proteins like BCL-2 and BCL-XL, have been correlated with resistance to ADCs like gemtuzumab ozogamicin [45] and brentuximab vedotin [46], indicating a potential mechanism of resistance to other ADCs like T-DM1, although this mechanism of resistance is not well defined in breast cancer.

#### 2.1.2. T-DM1 Combination Therapies

Current research strives to devise approaches to surmount these resistance mechanisms to improve patient prognosis. Personalized medicine strategies, including targeted and immune-based therapies, are under exploration to effectively counter resistance. In efforts to surmount resistance to T-DM1 in treating HER2-positive breast cancer, the integration of T-DM1 with diverse therapeutic agents has been extensively investigated. This strategy targets specific resistance mechanisms, proposing alternate modalities to boost treatment efficacy.

Many agents have been assessed for their potential synergistic effects with T-DM1, including monoclonal antibodies (e.g., pertuzumab), tyrosine kinase inhibitors (e.g., lapatinib, neratinib, tucatinib), PI3K pathway inhibitors (e.g., alpelisib), PD1/PDL1 checkpoint inhibitors (e.g., pembrolizumab, atezolizumab) as well as CDK4/6 inhibitors (e.g., palbociclib, ribociclib, abemaciclib) [47,48,49,50,51].

The logic behind these combinations is to mount a diversified onslaught on HER2-positive breast cancer cells, targeting various pathways and resistance mechanisms. Utilizing T-DM1 with these agents is anticipated to improve treatment results and confront the hurdles posed by drug resistance. Future clinical studies will shed light on the success of these combination treatments for patients with HER2-positive breast cancer.

#### 2.1.3. T-DM1 Toxicities and Safety Profile

With the development of new ADCs comes specific toxicities. In the landmark EMILIA trial [15], 15.5% of patients experienced a grade 3 adverse event (compared with 18% in the lapatinib–capecitabine arm). The most common grade 3 and 4 events with T-DM1 were thrombocytopenia (12.9%) and liver enzyme elevations of aspartate aminotransferase (4.3%) and alanine aminotransferase (2.9%). In the EMILIA trial, the occurrence of grade 3 or 4 thrombocytopenia was most common during the first two cycles of T-DM1 treatment, and the majority of patients were able to continue treatment with dose modifications. Overall, the incidence of bleeding was more common among patients treated with T-DM1 (29.8%) compared with lapatinib–capecitabine (15.8%), but rates of grade 3 or 4 bleeding were low in both groups (1.4% and 0.8%, respectively). One grade 4 gastrointestinal bleed did occur in a patient on T-DM1 but whose platelet count was within the normal range at that time. Additionally, most patients were able to continue treatment with dose modifications for elevated liver enzymes as well. These side effects highlight the importance of vigilant laboratory monitoring throughout the therapy [15,19,28].

Cardiotoxicity was also evaluated in the EMILIA trial, and the majority of patients treated with T-DM1 maintained an ejection fraction greater than or equal to 45% while on therapy (97.1%) [15]. Only one patient treated at the time of publication had developed grade 3 left ventricular systolic dysfunction after treatment with T-DM1. Baseline ejection fraction should be assessed and monitored throughout treatment while on T-DM1.

### 2.2. Trastuzumab Deruxtecan (T-DXd)

Trastuzumab deruxtecan (T-DXd) is a novel ADC that has transformed the treatment of breast cancer. T-DXd distinguishes itself by utilizing a topoisomerase I inhibitor derivative (i.e., deruxtecan) as its payload, connected via a cleavable tetrapeptide-based linker [20]. This cleavable linker is selectively severed within tumor cells, reducing off-target release and associated toxicities. T-DXd possesses a notably higher drug-to-antibody ratio (DAR) relative to T-DM1, an attribute that contributes to its potent efficacy [52].

Clinical investigations, including the pivotal DESTINY-Breast01 and DESTINY-Breast03 trials, have demonstrated T-DXd’s efficacy in significantly prolonging progression-free survival (PFS) over T-DM1, leading to its endorsement by the FDA as a second-line therapy for HER2-positive metastatic breast cancer [16,53]. Furthermore, T-DXd’s approval for HER2-Low breast cancer signals the recognition of a new subset within the breast cancer spectrum, expanding the therapeutic landscape [54].

While T-DXd has shown promising clinical efficacy in HER2-positive breast cancer, its use is accompanied by potential adverse effects that require vigilant monitoring. Paramount among these is the risk of drug-related interstitial lung disease (ILD) or pneumonitis, inflammatory conditions that may progress to severe impairment of lung function and significant respiratory compromise. Other toxicities include gastrointestinal symptoms, hematological abnormalities, and rare cardiac toxicities. Regular monitoring and appropriate supportive treatments are essential for managing these side effects [16].

#### 2.2.1. T-DXd: Mechanisms of Resistance

Trastuzumab deruxtecan (T-DXd) has emerged as a significant therapeutic agent in the treatment of HER2-positive and HER2-Low metastatic breast cancer. Nonetheless, resistance to T-DXd poses a significant obstacle in clinical settings. A thorough understanding of the resistance mechanisms is crucial to improving therapeutic strategies and subsequent patient outcomes.

1. HER2 Receptor Modifications: Modifications of the HER2 receptor are a primary resistance mechanism, including mutations, gene amplification, or structural alterations, which can diminish the receptor’s affinity for T-DXd. These modifications may reduce the drug’s efficacy by impairing target binding, necessitating the investigation of methods to overcome these changes in HER2 [55].
2. ADC Internalization and Intracellular Trafficking: The internalization and intracellular trafficking of T-DXd are critical to its cytotoxic action. Resistance may develop from disruptions in these processes, impeding the delivery of the cytotoxic payload. Enhancing T-DXd internalization and trafficking could be a strategic approach to bypass this resistance mechanism [56].
3. Drug-Efflux Transporters: The expression of efflux transporters, such as P-glycoprotein (P-gp), which expel the payload (deruxtecan) from cells, can decrease its intracellular concentration and cytotoxic impact. Inhibition or circumvention of these transporters is under investigation to enhance deruxtecan’s intracellular retention [34].
4. Tumor Microenvironment and Stromal Factors: The tumor microenvironment, including stromal cell-secreted factors, can confer survival advantages to cancer cells, fostering resistance to T-DXd. Targeting the tumor microenvironment through combination therapies or immune-modulating agents may address this resistance mechanism [57]. In the DAISY trial evaluating the efficacy of T-DXd in breast cancer patients at different levels of HER2 expression, researchers evaluated the impact of T-DXd on the tumor microenvironment. This exploratory study included samples collected from 31 patients. No significant changes in immune cell levels were observed at the 3-week or 6-week mark following treatment. However, a notable reduction in PD-L1 expression among the first cohort of patients was attributed to T-DXd’s cytotoxic effects on PD-L1-positive tumor cells. Additionally, a decline in the presence of macrophages close to tumor cells was reported in the same cohort [40].
5. Alternative Signaling Pathways: The activation of alternative signaling pathways, such as the PI3K/AKT/mTOR pathway, can provide survival advantages to cancer cells, undermining T-DXd’s effectiveness. Co-targeting HER2 and these alternative pathways may be essential to counteract resistance [7,43,58,59].
6. Tumor Heterogeneity: The intrinsic heterogeneity of tumors, both among patients and within a single tumor, can result in cancer cell populations with varying sensitivities to T-DXd, contributing to the complex nature of resistance. Personalized treatments, considering the unique molecular and phenotypic profiles of tumors, may be promising in overcoming resistance [60].

In summary, resistance to T-DXd in breast cancer is complex and involves diverse interactions between the tumor cells and their surrounding microenvironment. Current research is focused on elucidating these mechanisms in detail and developing targeted strategies to counter resistance. Anticipated advancements in T-DXd-based treatments for HER2-positive and HER2-Low breast cancer include personalized medicine, combination therapies, and an enhanced understanding of tumor biology.

#### 2.2.2. T-Dxd Combination Therapies

Therapeutic combinations are being rigorously evaluated to surmount resistance to T-DXd in breast cancer therapy. These research efforts aim to refine treatment regimens and enhance patient prognoses. A range of combinations are in various stages of clinical trials, following the same paradigm as T-DM1 above. The objectives of these clinical trials are to establish the safety profiles, efficacy, and appropriate dosing regimens for these combination treatments. The forthcoming results are expected to yield critical insights into the most efficacious combinations and patient demographics best suited for these therapies. The overarching aim is to personalize treatment approaches, thereby advancing the care and outcomes for patients with HER2-positive breast cancer who exhibit resistance to T-DXd.

#### 2.2.3. T-DXd in HER2-Low Breast Cancer

In the realm of breast cancer treatment, the categorization of tumors as HER2-positive or HER2-negative has been traditionally binary. However, a subset of tumors exhibit low levels of HER2 (“HER2-Low”), which is found in 45–60% of cases without HER2 amplification or overexpression [5]. These HER2-Low tumors are identified by an immunohistochemical (IHC) score of 1+ or a score of 2+ accompanied by a negative in situ hybridization (ISH) result. In the pivotal DESTINY-Breast 04 trial, trastuzumab deruxtecan (T-DXd) showed significant effectiveness in treating HER2-Low metastatic breast cancer, achieving a 52.6% objective response rate among patients who had undergone one or two prior lines of therapy [17].

While HER2-0 breast cancers are often less amenable to monoclonal antibody therapy, a subset known as HER2-Ultra-Low has been recognized, characterized by minimal HER2 protein expression. Ongoing studies are exploring the use of ADCs for this group. For example, the DESTINY-Breast06 trial is investigating the efficacy of T-DXd in patients with HER2-Ultra-Low metastatic breast cancer. Additionally, certain genetic mutations, like the V777L ERBB2 mutation and MutL deficiency—related to mismatch repair system changes—suggest potential responsiveness to anti-HER2 therapies, even in HER2-negative breast cancers.

Ongoing research is assessing T-DXd versus chemotherapy in hormone receptor-positive, HER2-Low metastatic breast cancer and exploring its combination with immune checkpoint inhibitors. Early data indicate favorable safety and efficacy, with high response rates [61]. The combination of T-DXd with immune therapies such as PD-L1 and PD-1 inhibitors is being evaluated, showing promising activity, although questions about the incremental benefit over T-DXd alone persist. These studies underscore the potential of T-DXd as a key therapeutic for HER2-Low breast cancer, offering hope for improved outcomes in this diverse patient population.

#### 2.2.4. T-Dxd Toxicities and Safety Profile

In the DESTINY-Breast03 trial, 45.1% of patients receiving T-DXd experienced any grade 3 or 4 adverse effects. The most common drug-related toxicities in the T-DXd arm were nausea (72.8%), fatigue (44.7%), and vomiting (44%). Overall, T-DM1 was better tolerated. Drug-related alopecia was also common, occurring in 36.2% of patients who received T-DXd (compared with 2.3% in patients treated with T-DM1). Neutropenia (19.1%), thrombocytopenia (7%), and leukopenia (6.6%) were also noted. Cardiotoxicity, as with T-DM1, appeared to be a rare event, but echocardiograms should be monitored.

The most concerning of the adverse events associated with T-DXd remains interstitial lung disease (ILD), or pneumonitis, which occurred in 10.5% of patients treated with T-DXd. The median time to onset of ILD/pneumonitis was 168 days (range: 33 to 507). Treatment was discontinued in 8.2% of patients treated with T-DXd due to pneumonitis. Fatal cases of ILD had occurred in earlier trials; however, most patients experience grade 1 or 2 events. Increased awareness has led to improved monitoring and treatment of this rare but serious side effect. Risk factors for the development of pneumonitis/ILD include dose >6.4 mg/kg, age <65, baseline oxygen saturation <95%, moderate to severe kidney impairment, pulmonary comorbidities (asthma, COPD, prior ILD/pneumonitis, pulmonary fibrosis, emphysema, and radiation pneumonitis) and >4 years since initial diagnosis [62].

Experts recommend high-resolution chest CT every 6 months for monitoring for pneumonitis and ILD in patients undergoing treatment with T-DXd, if available. If ILD is suspected, the drug must be held, and steroids should be promptly administered, in addition to pulmonary consultation and evaluation. If grade 1 toxicity occurs, the patient can be rechallenged with the drug. If grade 2 (symptomatic) toxicity occurs, the drug must be held permanently.

## 3. New HER2-Targeting ADCs on the Horizon

Emerging ADCs are being developed to enhance the therapeutic landscape of HER2-positive breast cancer treatments [63]. The advent of such ADCs has been revolutionizing the field, particularly by expanding the potential applications beyond traditional HER2-positive cancers to include tumors with lower expression levels of HER2 or with *ERBB*2 mutations. These novel ADCs are designed with modifications that optimize the delivery and release of cytotoxic agents within tumor cells, aiming to leverage the tumor microenvironment to enhance antitumor activity. Advancements in ADC technology are not only improving the effectiveness of these therapies but also aiming to reduce resistance and adverse effects. These improvements are the result of meticulous engineering that includes the selection of high-affinity antibodies, the development of potent payloads, and the creation of stable linkers that bind the payload until it reaches the tumor site.

The research and development of HER2 ADCs are expected to continue improving the therapeutic index, offering a broader spectrum of treatment options for patients with HER2-positive and HER2-Low breast cancer. These advancements signal a shift toward precision medicine, where treatment strategies can be personalized for better clinical outcomes and an enhanced quality of life for patients with breast cancer (Table 2).

### 3.1. Disitamab Vedotin (RC48)

Disitamab vedotin (DV), known as RC48, is a novel ADC directed against HER2-expressing cancer cells. The architecture of DV is characterized by a humanized anti-HER2 monoclonal antibody linked to the cytotoxic agent monomethyl auristatin E (MMAE) through a cleavable linker [70]. This design ensures that the monoclonal antibody binds selectively to the HER2 epitope, facilitating the internalization of MMAE, which then mediates cell death. DV’s binding affinity for HER2, which is higher than that of other therapeutic agents like trastuzumab, potentially increases its therapeutic impact. Furthermore, DV can induce cytotoxicity in adjacent cells—a phenomenon known as the bystander effect. Early clinical evaluations of DV have shown promising efficacy, particularly in patients with HER2 2+/ISH-negative status, indicating a potential for tailored therapy based on HER2 expression levels [71].

The safety and tolerability of RC48 have been evaluated in cancer patients in early-stage clinical trials. The most common mild adverse events (AEs) included fever, fatigue, and hematologic toxicity. The most common grade 3 or higher AEs were neutropenia, leukopenia, hypesthesia, and increased conjugated bilirubin levels. The incidence of serious AEs and dose-limiting toxicity were observed in the high-dose groups (2.5 mg/kg and 3.0 mg/kg); therefore, it appears the AEs of RC48 were dose dependent. Importantly, no drug-related pulmonary toxicity was seen with RC48 [25].

Disitamab vedotin and similar next-generation ADCs represent a significant advance in cancer therapeutics, combining precise tumor targeting with innovative payload mechanisms and improved linker technologies. These developments contribute to ADCs with variable DARs, refined safety profiles, and expanded potential for treating diverse cancer types and disease stages. It should be noted that DV is currently approved in China for gastric cancers [24].

### 3.2. ARX788

ARX788 represents an innovative ADC comprising an anti-HER2 monoclonal antibody, a non-cleavable linker, and a modified monomethyl auristatin F (MMAF), known as Amberstatin 269 (AS269) [22]. This ADC exhibits a DAR of 1.9. Preclinical studies, including those reported by Barok et al. in 2020, demonstrate ARX788’s superior efficacy over T-DM1 in trastuzumab-resistant breast cancer xenograft models [72]. A phase I trial showed ARX788 is well tolerated and has promising antitumor activity in patients with HER2-positive advanced gastric adenocarcinoma (ChinaDrugTrials.org.cn: CTR20190639) [73].

In the phase I ACE-Breast-01 study, the safety and antitumor activity of ARX788 was tested in patients with advanced HER2-positive breast cancer in China. The most common AEs occurring in more than 30% of patients were increased AST, increased ALT, corneal epitheliopathy, alopecia, hypokalemia, and ILD/pneumonitis (34.8%), which were mainly grades 1–2. Of note, grade 3–4 ILD/pneumonitis occurred in 2.9% of patients in this trial, and these were mainly late events after >100 days of treatment. Ocular toxicities were managed by a special task force and were primarily grades 1–2. Fortunately, all of these ocular AEs were reversible. Mild hypokalemia was an additional common toxicity (all grades 1–2) and was managed with oral potassium supplementation. Prolonged Qtc was observed in 20% of patients and recovered with observation in most cases. No drug-related deaths occurred. Based upon these results, the phase 2 recommended drug dosing was determined to be 1.5 mg/kg every 3 weeks [64].

Current phase II clinical trials are evaluating ARX788’s efficacy in various HER2-positive breast cancer contexts. Trial NCT05018676 is investigating its impact on HER2-Low breast cancers, while NCT05018702 focuses on patients with HER2-positive cancers with brain metastases. Additionally, NCT04829604, known as ACE-Breast-03, aims to determine the effectiveness of ARX-788 in patients with HER2-positive metastatic breast cancer who have previously undergone treatment with T-DXd [24].

### 3.3. Trastuzumab Duocarmazine (SYD985)

Trastuzumab duocarmazine (SYD985) is a novel ADC that combines trastuzumab with a cleavable valine–citrulline linker and a duocarmycin derivative, seco-DUBA, which is activated by proteases to cause DNA alkylation and cell death. It exhibits a bystander effect and has a DAR of 2.7 [21]. It showed efficacy in HER2-Low breast cancer models, surpassing T-DM1. In a phase 1 trial, it demonstrated antitumor activity with an objective response rate (ORR) of 28% for HR-positive and 40% for HR-negative metastatic breast cancer [24].

The phase III TULIP^®^ study compared SYD985 with physicians’ choice of treatment in participants with HER2-positive locally advanced or metastatic breast cancer. The trial met its primary endpoint, showing a statistically significant improvement in progression-free survival (PFS) for SYD985 over the physicians’ choice (7.0 months versus 4.9 months in favor of the trastuzumab duocarmazine group). PFS is the duration from randomization to disease progression or death from any cause. Additionally, the study reported preliminary supportive results for overall survival (OS) [69].

Safety data from this phase III trial were also collected. The most commonly reported adverse events were ocular events, including conjunctivitis (38.2%) and keratitis (38.2%), as well as fatigue (33.3%). Adverse events that led to discontinuation of the study drug were eye disorders (20.8%) and respiratory disorders (6.3%). There were no treatment-related deaths caused by trastuzumab duocarmazine [69].

### 3.4. BL-M0701

BL-M07D1 is a new ADC-targeting HER2 with a structure comprising the humanized antibody trastuzumab, a cathepsin B cleavable linker, and Ed-04, a camptothecin-derived topoisomerase I inhibitor. This inhibitor impedes the cell cycle in the S phase, inducing apoptosis. BL-M07D1 has a DAR of 8:1, akin to T-DXd, but with a more stable linker.

Preclinical evaluations using xenograft models revealed BL-M07D1’s superior tumor inhibition, outperforming T-DXd in low HER2-expressing models and both T-DM1 and T-DXd in HER2-positive models. Notably, BL-M07D1 demonstrated potent bystander effects, suggesting an enhanced efficacy against mixed HER2-positive/negative tumors [74]. These findings posit BL-M07D1 as a promising candidate in the treatment of a wider spectrum of breast cancers, surpassing the current HER2-targeting ADCs.

A phase I trial is ongoing in patients with metastatic breast cancer. The preliminary safety profile of BL-M07D1 from this study indicates that treatment-related AEs were mostly low-grade, with keratitis, anemia, and neutropenia being the most common. Grade 3 adverse events were noted at 42%, with anemia (23%) and neutropenia (17%) being more prevalent. There was one case of grade 4 neutropenia. No dose-limiting toxicities were reported at the recommended dose, and there were no treatment-related deaths [75]. Further studies are needed to fully assess the safety and efficacy of BL-M07D1.

### 3.5. Zanidatamab Zovodotin

Zanidatamab zovodotin, also referred to as ZW49, is an innovative bispecific ADC aimed at treating HER2-expressing or HER2-amplified cancers, including breast cancer. It is currently undergoing clinical evaluation to determine its efficacy and safety profile. The first-in-human phase I trial was designed to determine the maximum tolerated dose, characterize its safety and tolerability, and evaluate its antitumor activity as monotherapy [26]. The phase 1 study of zanidatamab zovodotin (ZW49) in patients with HER2-positive solid cancers found it to have an acceptable safety profile, with the majority of AEs being low-grade and manageable. The most common treatment-related AEs included keratitis, alopecia, and diarrhea, primarily of grade 1 or 2 severity. No dose-limiting toxicities were observed for the selected dosing regimens in the dose-escalation phase, and no treatment-related deaths occurred. Among eight patients with breast cancer, zanidatamab zovodotin achieved a confirmed ORR of 13%, which included a partial response (PR) rate of 13% and a stable disease (SD) rate of 38%. The recommended dose for further studies was identified as 2.5 mg/kg every 3 weeks, showing promising antitumor activity in heavily pretreated patients [76].

### 3.6. Other ADCs in Clinical Trials

There are many other ADCs in clinical trials, including ALT-P7, SHR-A1811, and TQB2102 (Table 2). The safety and therapeutic benefit of these molecules will be determined by the results of these trials and additional future clinical trials.

## 4. Future Directions

Antibody–drug conjugates (ADCs) represent a sophisticated class of therapeutic agents that have transformed the treatment landscape for HER2-positive and HER2-Low breast cancer. The successful development of novel HER2-targeted ADCs will require not only advancements in technology but also predictive preclinical models and well-designed clinical trials.

The conjugation process, particularly site-specific conjugation, is at the forefront of ADC technology advancements [22]. Traditional conjugation methods may result in heterogeneous ADC populations, leading to variability in efficacy and safety. The industry trend is shifting toward site-specific conjugation methods to produce more uniform ADCs, which could lead to improved therapeutic outcomes. Site-specific conjugation involves precise and consistent attachment of payloads to the antibodies, ensuring a uniform drug-to-antibody ratio (DAR), which is a critical quality attribute of ADCs [77]. Several methods facilitate site-specific conjugation. Antibody engineering techniques such as THIOMABs utilize engineered cysteine residues in antibodies to attach payloads at specific sites. Chemical methods, like AJICAP, leverage distinct functional groups on antibodies for consistent conjugation. Enzymatic methods offer another layer of specificity by using enzymes that recognize specific peptide or carbohydrate motifs on antibodies for payload attachment. Among the most exciting advancements are Tag-free enzymatic methods that negate the need for engineered tags on antibodies, simplifying the process and potentially improving the manufacturability and scalability of ADC production [78,79,80]. These methods utilize naturally occurring glycan sites on antibodies for the attachment of payloads using glycan-specific enzymes, allowing for a homogeneous product without the need for genetic modification of the antibody. In a recent publication, Fujii et al. [81] introduced the AJICAP method, a novel “second-generation” technology designed to precisely link therapeutic agents to antibodies. This method circumvents the commonly encountered issue of protein aggregation during such conjugation processes. Unlike traditional methods that necessitate altering an antibody’s structure through redox treatments, AJICAP simplifies the process by utilizing a “one-pot” reaction that maintains the antibody’s integrity. This innovation significantly bolsters the stability of the compounds involved in coupling drugs to antibodies, which facilitates the generation of antibody–drug conjugates (ADCs) with a uniform and exact drug-to-antibody ratio. The team has refined the process to enable dual-site drug attachment on the antibody, resulting in the synthesis of over 20 distinct ADC formulations. The versatility of the AJICAP technology is further demonstrated by its application in formulating other conjugate types, such as antibody–protein and antibody–oligonucleotide conjugates. The advancements offered by this technique hold the promise for the creation of ADCs that are not only more efficacious but also safer for oncological applications, all without the necessity for genetic modification of the antibodies. The evolution of these component technologies demonstrates a deliberate move toward precision in ADC design, aiming to enhance efficacy, reduce off-target effects, and improve patient outcomes. A comprehensive understanding of these technologies is essential to advancing the field and optimizing the therapeutic potential of ADCs.

Although efficacy is the main goal in the development of ADCs, ensuring their safety profile is essential. Preclinical models are not infallible in predicting human toxicity, which can lead to unexpected challenges during clinical trials. For instance, DHES0815A, an ADC that initially demonstrated considerable promise with its high effectiveness and apparent tolerability in preclinical assessments, encountered a significant setback. Despite the encouraging preclinical results, it exhibited severe dermatological, ocular, and pulmonary toxicities in the first phase of human trials, resulting in its failure to proceed further [82]. This discrepancy underscores the need to delve into the potential causes behind such unpredicted outcomes and to strategize on enhancing the predictive accuracy of preclinical models for ADC development.

**Figure 3:**
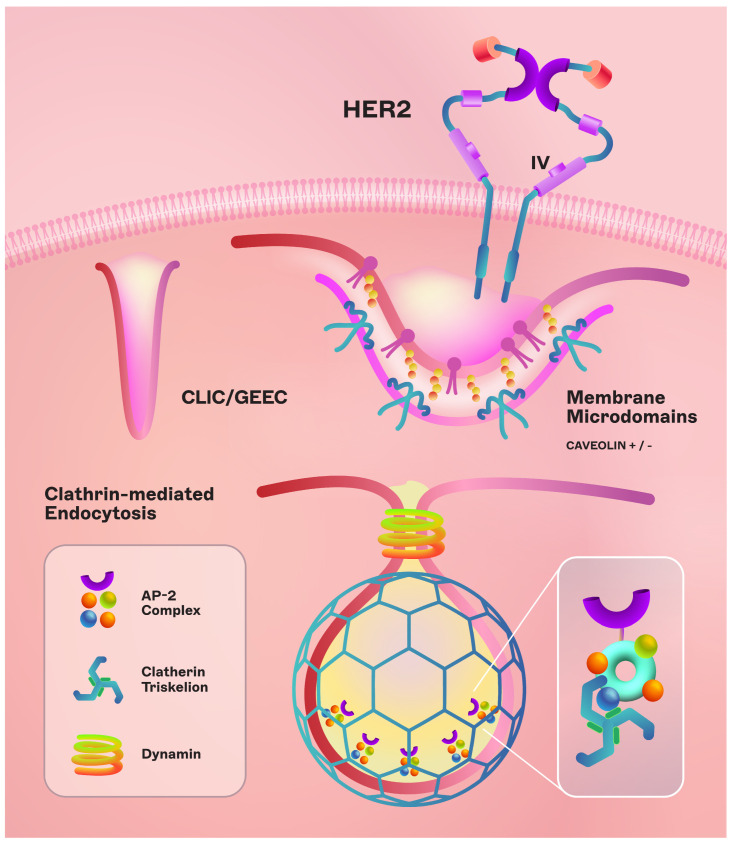
ADC internalization and targeting specificity. IV refers to Domain IV of the extracellular domain of the HER2 protein.

There are numerous biological obstacles that arise in ADC clinical development, including resistance mechanisms such as antigen downregulation and impaired drug internalization (Figure 3). Novel strategies like alternative linkers and checkpoint inhibitors are being explored to tackle these challenges. Pharmacokinetic and pharmacodynamic optimization are pivotal for ensuring that therapeutic drug levels are maintained with minimal toxicity. Additionally, technical and manufacturing complexities, such as intricate production processes and limited manufacturing capabilities, pose significant barriers. Regulatory frameworks also demand rigorous adherence to ensure ADC quality and safety. Financial constraints further complicate ADC development due to the costly nature of their specialized production. To surmount these challenges, strategies such as the selection of preclinical models mirroring human pathology, identification of predictive biomarkers, integrated PK/PD modeling, and iterative learning from clinical trials are essential.

Additionally, the exploration of ADC sequencing for disease progression and the combination with other targeted therapies hold promise for overcoming resistance and enhancing treatment efficacy. Tailoring treatments based on individual molecular and genetic profiles, facilitated by next-generation sequencing, signifies a shift toward more personalized and potent combination therapies in cancer treatment.

## 5. Conclusions

HER2-positive breast cancer remains an aggressive disease with historically poor outcomes. HER2-targeted ADCs have emerged as a promising strategy for breast cancer treatment, with a particular focus on enhancing outcomes for both patients with HER2-positive and HER2-Low breast cancer. The development of T-DM1 and T-DXd has significantly improved patient outcomes, but resistance and progression still occur. Additionally, with these novel drug mechanisms come additional side effects and toxicities that must be carefully monitored, specifically thrombocytopenia and elevated liver function enzymes for T-DM1 and gastrointestinal effects, alopecia, and pneumonitis for T-DXd.

In this review, we provide an updated summary of the current state of approved HER2-targeted ADCs as well as a detailed review of selected investigational agents on the horizon. We have discussed the characteristics and available efficacy and safety data on the novel molecules RC48, ARX-788, SYD985, BL-M0701, and the bispecific ADC zanidatamab zovodotin (ZW25). Clinical trials are crucial in determining the optimal dosing regimens, understanding resistance mechanisms, and identifying patient populations that would derive the most benefit from these treatments. With a focus on innovation and precision, these novel ADCs are at the forefront of a new era in targeted cancer therapy, holding the potential to improve outcomes for patients with HER2-positive and HER2-Low breast cancer.

The exploration of novel sequences and combinations incorporating ADCs represents another promising frontier in cancer therapy. These combinations have the potential to expand the therapeutic reach, overcome resistance mechanisms, modulate the immune system, and offer tailored treatments to specific patient subgroups. Ongoing clinical trials and research efforts are essential in advancing our understanding of optimal sequential approaches and combinations and their potential to improve patient outcomes in the complex landscape of cancer treatment.

## Figures and Tables

**Table 1:** Key characteristics of selected HER2 ADCs.

ADC Name	mAb	Payload	Linker	DAR	Clinical Phase	References
Trastuzumab emtansine (T-DM1)	Trastuzumab	DM1	Non-cleavable SMCC linker	3.5	Approved for metastatic HER2-positive breast cancer, residual disease after neoadjuvant therapy	[18,19]
Trastuzumab deruxtecan (DS-8201a)	Trastuzumab	DXd	Cleavable GGFG linker	8	Metastatic HER2-positive and HER2-Low breast cancer	[16,20]
Trastuzumab duocarmazine (SYD985)	Trastuzumab	seco-DUBA	Cleavable vc linker	2.7	Phase I/II—advanced breast cancer	[21]
ARX-788	Anti-HER2 mAb (ARX269)	MMAF	Non-cleavable linker conjugated to pAcF	1.9	Phase II—advanced breast cancer	[22]
ALT-P7	Trastuzumab biobetter (HM2)	MMAE	Cleavable cysteine-containing peptide	2	Phase I	[23]
BL-M07D1	Trastuzumab	Ed-04	Cathepsin B cleavable linker	8	Phase I—advanced breast cancer	[24]
Disitamab vedotin (RC48)	Hertuzumab	MMAE	mc-val-cit PABC linker	4	Phase I	[25]
Zanidatamab zovodotin	Zanidatamab	Zovodotin	Cleavable vc linker	2–4 (variable)	Phase II	[26]

Abbreviations: mAb: monoclonal antibody; DAR: drug-to-antibody ratio; DM1: derivative of maytansine 1; SMCC: succinimidyl 4-(*N*-maleimidomethyl) cyclohexane-1-carboxylate; DXd: derivative of exatecan; GGFG: glycine–glycine–phenylalanine–glycine; vc: valine–citrulline; seco-DUBA: seco-duocarmycin hydroxybenzamide azaindole; MMAF: monomethyl auristatin F; pAcF: para-acetylphenylalanine; MMAE: monomethyl auristatin E; mc-val-cit PABC: maleimidocaproyl–valine–citrulline–p-aminobenzylcarbamate.

**Table 2:** Clinical trials of investigational antibody–drug conjugates for HER2-positive breast cancer.

ADC	Study Title	Key Eligibility	Primary Endpoint	Phase	N	Efficacy and Results	References
ALT-P7 (HM2-MMAE)	Clinical Study of ALT-P7 to Determine Safety, Tolerability and Pharmacokinetics in Breast Cancer Patients [NCT03281824]	HER2-positive MBC	DLT, MTD	I	27	ORR: 77% mPFS: 6.2 months	[23]
ARX788	Phase I Trial of a Novel Anti-HER2 Antibody-Drug Conjugate, for the Treatment of HER2-Positive Metastatic Breast Cancer	HER2-positive MBC	Safety, pharmacokinetics, and antitumor activity	I	69	ORR: 65% DCR 100% mPFS 17.02 months	[64]
ARX788	Phase 1 Dose Escalation Study of ARX788, a Next-Generation Anti-HER2 Antibody Drug Conjugate, in Heavily Pretreated Breast Cancer Patients [ACE-PanTumor-01 trial (ARX788-1711; NCT03255070]	HER2-positive and HER2-Low MBC	Safety, pharmacokinetics, and antitumor activity	I	42	HER2-positive ORR 36% HER2-Low ORR 17%	[65]
ARX788	Efficacy and Safety of Pyrotinib Maleate Combined with ARX788 Neoadjuvant Treatment in Breast Cancer Patients [NCT04983121]	Stage II-III HER2-positive breast cancer patients experiencing a poor efficacy of trastuzumab and pertuzumab	Residual tumor burden (RCB)	II	30	N/A	Recruiting
ARX788	ARX788 in HER2-positive, Metastatic Breast Cancer Subjects (ACE-Breast-03) [NCT04829604]	HER2-positive MBC previously treated with T-DXd	ORR	II	71	ORR: 57%	[66]
ARX788	ARX788 in HER2-positive Breast Cancer Patients with Brain Metastases [NCT05018702]	HER2-positive, MBC-resistant, or refractory to tyrosine kinase inhibitors (TKI)	Central nervous system (CNS) clinical benefit rate (CBR)	II	32	N/A	Recruiting
ARX788	ARX788 in Breast Cancer with Low Expression of HER2 [NCT05018676]	HER2-Low MBC	ORR	II	54	N/A	Recruiting
BL-M07D1	A Study of BL-M07D1 in Patients with Locally Advanced or Metastatic HER2 Positive Breast Cancer and Other Solid Tumors [NCT05461768]	Locally advanced or metastatic HER2-positive/low-expression breast cancer and other solid tumors	DLT, MTD	I	15	HER2-positive MBC-ORR 60%	[67] (Recruiting)
Disitamab vedotin (RC48)	RC48-ADC, a HER2-targeting antibody-drug conjugate, in patients with HER2-positive and HER2-low expressing advanced or metastatic breast cancer: A pooled analysis of two studies [NCT02881138; NCT03052634]	HER2-positive metastatic solid tumors	ORR	I	118	HER2-positive ORR 42.9%, mPFS 6.3 months HER2-Low ORR 39%, mPFS 5.7 months	[68]
SYD985 vs. PC	SYD985 vs. Physician’s Choice in Participants with HER2-positive Locally Advanced or Metastatic Breast Cancer (TULIP) [NCT03262935]	HER2-positive MBC	PFS	III	437	mPFS SYD985: 7 months PC: 4.9 months	[69]
TQB2102	A Study of TQB2102 for Injection in Patients with Recurrent/Metastatic Breast Cancer [NCT06115902]	HER2-positive MBC	Toxicity, ORR	I	150	N/A	Recruiting
XMT-1522	Study of Antibody Drug Conjugate in Patients with Advanced Breast Cancer Expressing HER2 [NCT02952729]	HER2-positive MBC	Dose escalation/objective response	Ib	120	Discontinued due to toxicity	[23]

Abbreviations: MMAE: monomethyl auristatin E; DLT: dose-limiting toxicity; MTD: maximum tolerated dose; RCB: residual cancer burden; T-DXd: trastuzumab deruxtecan; ORR: objective response rate; DCR: disease control rate; TKI: tyrosine kinase inhibitor; CNS: central nervous system; CBR: clinical benefit rate; mPFS: median progression-free survival; PC: physician’s choice; mo: months; N/A: data not available.

## Data Availability

Not applicable.
